# Clinical and economic impact of the availability of innovative therapies for advanced lung cancer in men in Latin America: a population-based secondary data study

**DOI:** 10.1016/j.lana.2025.101172

**Published:** 2025-07-02

**Authors:** Andrés F. Cardona, Natalia Sánchez, Liliana Gutiérrez-Babativa, Leonardo Rojas, Jairo Zuluaga, Stella Martínez, Lucia Viola, Carlos Carvajal, Juliana Bogoya, Laura Prieto-Pinto, Daniel Samacá-Samacá, Antonio Robles, Joshua Kock, Claudio Martín, Luis Corrales, Luis E. Raez, Vladmir Cordeiro de Lima, Suraj Samtani, Oscar Arrieta

**Affiliations:** a Institute of Research, Science and Education, Luis Carlos Sarmiento Angulo Cancer Treatment and Research Center (CTIC), Bogotá, Colombia; b GIGA/TERA Research Groups (CTIC/Universidad El Bosque), Bogotá, Colombia; c Thoracic Oncology Unit, Luis Carlos Sarmiento Angulo Cancer Treatment and Research Center (CTIC), Bogotá, Colombia; d Clinical Oncology Unit, Instituto Nacional de Cancerología – INC, Bogotá, Colombia; e Evidence Generation Team, Roche Colombia, Bogotá, Colombia; f Thoracic Oncology Unit, Instituto Alexander Fleming, Buenos Aires, Argentina; g Medical Oncology Unit, Centro de Investigación y Manejo del Cáncer (CIMCA), San José, Costa Rica; h Thoracic Oncology Department, Memorial Cancer Institute, Memorial Health Care System, Florida Atlantic University (FAU), Miami, FL, United States; i Thoracic Oncology Reference Center, A.C. Camargo Cancer Center, São Paulo, Brazil; j Clínica las Condes Cancer Center, Santiago de Chile, Chile; k Thoracic Oncology Unit, Instituto Nacional de Cancerologia (INCan), Mexico City, Mexico

**Keywords:** Lung neoplasms, Drug approval, Innovate therapies, Latin America

## Abstract

**Background:**

Over the last decade, the development of innovative cancer treatments has accelerated and has been associated with improved mortality trends; however, local regulatory approval times are extensive. This study estimated the clinical and economic impact of delays in the approval of innovative therapies for the treatment of advanced lung cancer in men in five Latin American countries.

**Methods:**

Using public data, we estimated the relationship between available innovative therapies (AIT) and age-specific mortality rate (ASMR) for Argentina, Brazil, Chile, Colombia, and Mexico through a regression model. Based on the difference between the number of FDA-approved therapies and the number approved by each local agency, we calculated the avoidable deaths (ADs) if innovation had been available. We estimated the Years of Life Lost (YLLs) using the life expectancy, the median age of death, and the ADs. Productivity loss (PL) was calculated using each country’s retirement age and yearly Gross Domestic Product per capita (GDPc) in 2022 constant USD.

**Findings:**

Total ADs, YLLs, and PL were 8694, 114,477, and USD 439,179,876, respectively. Argentina had the highest impact of AIT on ASMR. Brazil’s results showed a high clinical and economic impact, primarily due to its large population. Chile’s high GDPc led to high PL. Colombia and Mexico showed a high clinical impact, suggesting a benefit of early approval. Differences in availability and approval times have increased with the number of FDA-approved therapies, yet local time gaps have recently increased.

**Interpretation:**

Our study shows the substantial clinical and economic impact of delays in approving innovative therapies, underscoring the potential of improving regulatory processes to increase the availability of lung cancer treatments. Accelerating the introduction of innovative therapies for advanced lung cancer in Latin America represents a significant opportunity to enhance survival rates, instilling hope and optimism while also avoiding substantial PL.

**Funding:**

This study was conducted as a research partnership between Roche and CTIC. No funding was received. Authors participated in the study design, data collection, data analysis, interpretation, and writing of the report.

**Research in context**

**Evidence before this study**

We conducted a comprehensive literature search across PubMed, Google Scholar, and regulatory agency repositories to identify studies reporting on the impact of delayed approval of innovative therapies, particularly in the context of Latin America. We included English and non-English publications up to October 2024, thereby ensuring a broad inclusion of relevant evidence. Our search terms included “innovative therapies lung cancer”, “regulatory approval delays”, “cancer mortality reduction”, and “economic impact of cancer treatment”. Our search focused on studies detailing the economic and clinical consequences of delayed cancer therapy approvals and methodological frameworks for measuring such impacts. While some studies have demonstrated the positive impact of innovation, with reductions of more than one million cancer deaths in the United States correlated with the approval of cancer treatments, or specifically in lung cancer, an increase in survival associated with the timely approval of targeted therapies, this has not been assessed in Latin American countries. Consequently, although reports such as FIFARMA’s W.A.I.T. indicator highlight the barriers and lengthy approval and access times for innovative treatments in Latin America, the correlation between these approval delays within the region and their impact on clinical and economic outcomes in oncology remains unevaluated. The findings of this search supported the study’s rationale, the most fitting methodological approach, and the data sources used.

**Added value of this study**

This study contributes to the understanding of the clinical and economic implications of delayed approvals for innovative treatments for lung cancer in five Latin American countries: Argentina, Brazil, Chile, Colombia, and Mexico. While existing literature focuses on the benefits of timely approvals in high-income countries, our research addresses a significant gap by providing regional data and insights into the regulatory landscape in Latin America, where delays in approving innovative therapies are extensive, limiting the population’s timely access to effective treatments. We estimate the number of deaths, years of life lost, and productivity loss that can be avoided, offering a comprehensive view of the economic and health benefits associated with timely access to innovative therapies. Moreover, our findings highlight the variability in approval times and the consequent impacts across the region. This evidence is crucial for shaping local policies that aim to improve regulatory efficiency in emerging economies.

**Implications of all the available evidence**

The combined evidence underscores the pressing need for expedited regulatory processes to ensure timely access to innovative cancer treatments, particularly in Latin American countries. Our findings advocate for policy reforms aimed at minimising approval delays to significantly reduce lung cancer mortality and associated economic burdens. Future research should focus on implementing and assessing the effectiveness of strategies that streamline approval processes, such as regulatory reliance and accelerated pathways, as well as identifying and overcoming specific local barriers to adopting such mechanisms across the diverse healthcare systems in Latin America. These findings should inform healthcare policymakers in prioritising strategies to expedite approval mechanisms and increase the accessibility of innovative therapies, ultimately improving patient outcomes and economic productivity.

## Introduction

Cancer imposes a considerable burden worldwide. In 2022, there were 19 million new cancer cases and 9·7 million cancer deaths worldwide. In Latin America and the Caribbean, there were 1·5 million new cases and 750,000 cancer deaths.^1^ Cancer results in an economic burden for patients, healthcare systems, and countries due to healthcare spending and productivity losses from morbidity and premature mortality. In the US, the financial burden of cancer is approximately 1·8% of gross domestic product (GDP). In 2017, estimated cancer healthcare spending was US$161·2 billion; productivity loss from morbidity was US$30·3 billion; and premature mortality was US$150·7 billion.^2^ This high burden of morbidity has driven numerous efforts to combat cancer, both in terms of funding cancer research and funding to cover cancer medications.^3^^,^^4^ Large outlays from both public and private sectors may be starting to pay off, as cancer mortality has been declining in recent years. Between 2000 and 2010, global age-adjusted cancer mortality rates decreased by around 1% annually worldwide.^3^

Access to new medications is always pressing for patients, physicians, and healthcare system managers. Previous studies have suggested that new pharmaceutical treatments were responsible for 10–30% of cancer mortality reductions between 1990 and 2011^5^ and 24% between 2000 and 2016 in the United States.^3^ Several studies conducted in different countries have also revealed that the number of newly approved treatments was associated with significant reductions in premature mortality and that these survival gains were highly cost-effective.^3^ The introduction of new cancer treatments has played a significant role in reducing cancer mortality and led to significant advances in the treatment paradigm for certain types of solid and haematological tumours. In the last decade, there has been a considerable push to develop new cancer drugs, with approximately 184 new oncology therapies introduced worldwide between 2002 and 2021.^6^

MacEwan et al. found that changes in the number of cancer drugs approved between 2000 and 2016 correlated with significant reductions in mortality in the 15 most common tumours in the US and were associated with 1·3 million cancer deaths avoided during this period.^3^ Improvements in mortality were more significant in the most common tumour types with relatively more approvals, such as lung cancer, colorectal cancer, breast cancer, melanoma, lymphoma, and leukaemia, highlighting the need to continue innovating in oncology treatments, particularly in tumour types with few existing approvals.^3^ Lung cancer is the leading cause of cancer-related mortality worldwide, accounting for 1,817,469 deaths in 2022, representing an age-standardised rate of 16·8 per 100·000 people.^7^ Despite the increase in lung cancer incidence among females, the male population continues to be the most affected, with incidence and mortality rates two times higher in men than women,^8^ becoming the leading cause of cancer death in men in 93 countries.^9^

The relationship between advances in lung cancer treatment and their effect on mortality has been previously evaluated.^10^ Howlader et al. found that, in patients with non-small lung cell cancer (NSCLC), mortality decreased faster than incidence, with a change in survival rates from 26% in patients diagnosed in 2001 to 35% in patients diagnosed in 2014, which the authors related to the positive effect of timely approval of targeted therapies on survival.^10^

Most cancer medications enter the US market first. Approvals of new oncological drugs by the Food and Drug Administration (FDA) can influence regulatory decisions in other regions.^11^ However, patients’ access to innovative medicines is often significantly delayed due to lengthy approval times and may substantially impact patients’ survival and quality of life. This is especially true in cancer patients, both in the metastatic context (given their reduced or limited life expectancy) and in the curative context (where adjuvant medications improve the chances of cure).^12^^,^^13^ A treatment delay of four weeks can increase cancer-related mortality.^13^ Quality of life is also negatively affected by delays that hinder timely access to new therapies capable of prolonging survival, slowing disease progression, and alleviating cancer-related symptoms. Delays in access are regrettable, given the increasing effectiveness of new therapeutic advances, which prolong patients’ lives but remain inaccessible to those who need them.^14^

The availability of innovative drugs in Latin America is particularly concerning. The Latin American Pharmaceutical Industry Federation (known in Spanish as FIFARMA) conducted a study to assess the regional availability and access to 288 innovative therapies globally approved by 2021.^15^ The FIFARMA report shows that regulatory approval of oncology drugs in Latin America takes, on average, 2 years after global approval.^15^ According to this report, Brazil, Argentina, Mexico, and Chile have the fastest approval times in the region (approximately 2 years); in contrast, Colombia has the longest regulatory approval timeline, with more than 3 years after global approval.^15^ The FIFARMA initiative emphasises that improving the availability of innovative medicines in Latin America is a priority for all actors in the health system.^15^^,^^16^ However, although this study clearly shows the time to approval at the regional level, it does not evaluate the impact of these delays at the clinical or economic level.

In response to this issue, regional information must be generated to guide the development of local regulatory policies, prioritising and organising oncology services through research into the association between delays in approving oncologic therapies and mortality. This study aims to estimate the clinical and economic impact of the availability of innovative treatments for treating advanced lung cancer in Colombia, Chile, Argentina, Brazil, and Mexico by analysing changes in age-specific mortality and productivity loss.

## Methods

This was an analytical observational study using secondary data retrieved from public sources. The analysis covered information from the first FDA-approved innovative therapy until 2021 and was conducted for five Latin American countries: Argentina, Brazil, Chile, Colombia, and Mexico. Considering that the term “innovative” is a concept that evolves over time, we included, as innovative therapies, treatments developed to improve patient outcomes and reduce side effects compared to conventional approaches. The first monoclonal antibody targeting VEGF was bevacizumab, approved by the FDA for lung cancer in 2006.^17^ Additional FDA-approved therapies, such as immunotherapy or targeted agents, indicated for treating lung cancer were also included.

Clinical practice guidelines were used to identify innovative therapies for treating advanced lung cancer. The approval dates of the FDA and local regulatory agencies were retrieved from public records, including drug approval search engines and official documents (e.g., minutes, records, resolutions), as well as the official websites of governmental bodies, regulatory agencies, and ministries of health for each country. The local regulatory agencies searched were ANMAT (Argentina), ANVISA (Brazil), ANAMED and the Institute of Public Health (Chile), INVIMA (Colombia), and COFEPRIS (Mexico).

Local approval times for oncology treatments in Latin American countries have been reported to take an average of two years after FDA approval.^15^ A preliminary analysis of data on the FDA and the local agencies’ approval dates showed that, in some cases, local approval may occur in the same year as FDA approval. This finding is consistent with studies on the regulatory landscape and registration pathways of innovative therapies in the region, which have shown that some countries have begun implementing strategies to accelerate local approval times.^18^^,^^19^ For this reason, one year after FDA approval was defined as the average assessment time for local approval, with longer approvals considered delayed.

We conducted a multivariate regression to estimate the effect of the number of indications approved in each country on the annual age-specific mortality rate (ASMR), adjusting for the incidence rate. The ASMR for trachea, bronchus, and lung cancer reported by WHO was retrieved from the Mortality Database (up to 2021).^20^ The model was performed using the ASMR on men due to the historically high burden of disease in this population compared to women. The age-specific incidence data, used as the control variable in the model, was retrieved from the Institute for Health Metrics and Evaluation (IHME), specifically, the results of the 2019 Global Burden of Disease (GBD) study.^21^

Including incidence data allowed for controlling the impact of possible effect modifiers that might affect the model estimation. Data for age-specific incidence rates was available up until 2019. Imputation for 2020 and 2021 was conducted, considering the stable trend in the incidence rate from the last years in all included countries. For the imputation of each year, the average rate of the previous five years was employed; for example, to impute the missing data for 2020, the average incidence rate between 2015 and 2019 was used. The same was applied for 2021. Based on the regression model results, we calculated the number of avoidable deaths, years of life lost, and productivity loss if the innovative therapies had been approved on time in each country, e.g., one year after FDA approval. These estimates required country-level information on the adult population by age group (only available for all countries until 2021), including life expectancy (at birth and age 60), annual gross domestic product (GDP) per capita, and average retirement age. This information was extracted from the United Nations Statistics Division and The World Bank.

### Statistical analysis

#### Impact of innovation on lung cancer mortality

First, the number of treatments and indications available for advanced lung cancer in each country for each year was identified according to the local regulatory approval dates. To estimate the effect of the available innovative therapies for treating advanced lung cancer on lung cancer mortality, we adapted Lichtenberg’s methodology^22^, ^23^, ^24^, ^25^ by using the following multivariate regression model for each country (formula a):

(a) $${ASMR}_{t}=\alpha \;+\;\beta_{1}TA_{t}\;+\;\beta_{2}inc_{t}\;+\;\epsilon_{t}$$

where ASMR_t_ represents the age-specific mortality for each age group in each country for each year; TA_t_ the number of treatments available for advanced lung cancer in each country for each year; inc_t_ the age-specific incidence rate in each country for each year; and ϵ_t_, the error term. The estimated coefficient for $\beta_{1}$ was defined as the effect on the ASMR of the actual number of available advanced lung cancer innovative therapies in each country.

We calculated the difference between the number of therapies and indications approved by the FDA and the number approved by each local agency each year, granting one year for local assessment, reflecting the local delay in the availability of innovative therapies and indications for advanced lung cancer. These differences were then multiplied by the estimated effect $\beta_{1}$ to obtain the total associated effect on the ASMR (Total Effect). That is the total effect the innovation would have had on lung cancer mortality if all treatments and indications were available during that year.

#### Avoidable deaths

The number of deaths from lung cancer was estimated based on the ASMR reported by WHO and the total population of each five-year age group. Then, the Total Effect was subtracted from the ASMR to obtain a simulated ASMR representing a mortality scenario with all available innovations (Formula b).

(b) SimulatedASMR=ASMR−TotalEffect

The simulated ASMR was multiplied by the population of each age group to estimate a simulated number of deaths by year. The difference between the actual deaths (based on the ASMR reported by WHO) and the estimated deaths, if those treatments and indications had been available, provided the number of age-specific avoidable deaths (AD) (formula c).

(c) AD=lungD−SimulatedlungD

#### Years of life lost

We calculated the years of life lost (YLL) by estimating the difference between the median age of death in each age group ([15–19 years = 17 years], [20–24 years = 22 years] … [80–84 years = 82 years]) and the life expectancy at birth (for those who died under 60 years) or the life expectancy at 60 (for those who died over 60 years). This result was multiplied by the number of age-specific avoidable deaths to obtain the total YLLs in each country for each year.

(d) yearsoflifelost=ADx(lifeexpectancy−medianageofdeath)

#### Productivity loss

Productivity loss was obtained by multiplying the total YLLs by each country’s gross domestic product (GDP) per capita for each year. To estimate the years of productive life lost (YLLp), only the YLLs up to the retirement age of each country were considered (formula e).

(e) productivityloss=YLLpxGDPpercapita

First, we assessed the robustness of the model by using the 95% confidence interval of the $\beta_{1}$ obtained from the regression model to estimate the possible variability in clinical and economic impact. The second sensitivity analysis assessed the use of imputed age-specific incidence data for 2020 and 2021 based on the average value of the last 5 years. In this sensitivity analysis, instead of using the average of the previous years, we used the last reported value for each age group, corresponding to 2019. Results were reported by country. The financial impact was reported in constant 2022 US dollars, calculated from the constant 2015 GDP reported by the World Bank.

Statistical analyses were performed in Stata (version 14).

The study was conducted using publicly available sources with no patient information. No informed consent or ethics committee was required.

### Role of the funding source

This study was conducted as a research partnership between CTIC and Roche’s evidence generation team; there was no funding. Authors participated in the study design, data collection, data analysis, interpretation, and writing of the report.

## Results

The FDA approved the first innovative therapy for lung cancer in 2006. It was considered possible that local approval occurred in the same year as FDA approval; therefore, the analysis was conducted from 2006 onwards. It was established that the expected time for local submission and approval was the same year or one year after FDA approval. Local approvals later than one year after FDA approval were considered regulatory delays. Considering that the latest data by age group is available up to 2021, only therapies approved by the FDA up to this year were included. During the analysis period, 24 FDA-approved innovative treatments for advanced or metastatic lung cancer were identified, with 30 indications. By 2021, however, Argentina had 21 (70%) of FDA-approved indications available, Brazil 22 (73%), Chile 23 (77%), Colombia 18 (60%), and Mexico 20 (67%).

Colombia has faced long approval times since 2015. Local approval for new molecular entities has taken over three years, and in some cases up to five years following FDA approval. In Argentina, approval times were generally short; however, between 2019 and 2020, there were cases of local approvals three years after the FDA decisions. In Brazil, the first approvals of innovative therapies for advanced lung cancer presented extensive approval times, ranging from three to five years after FDA approval. However, since 2016, an average of one to two years for local approval has been identified.

Short local approval times were identified in Mexico, between one and two years after FDA approval, with sporadic cases up to three years. These few cases with longer approval times are not associated with a specific period or whether they were new molecules or new indications for molecules already approved. Finally, in Chile, more significant variability in approval times was identified, with several new molecules or new indications approved locally the same year the FDA approved them, and other cases with three or up to five years’ difference in local approval after the FDA approval.

**Fig. 1:**
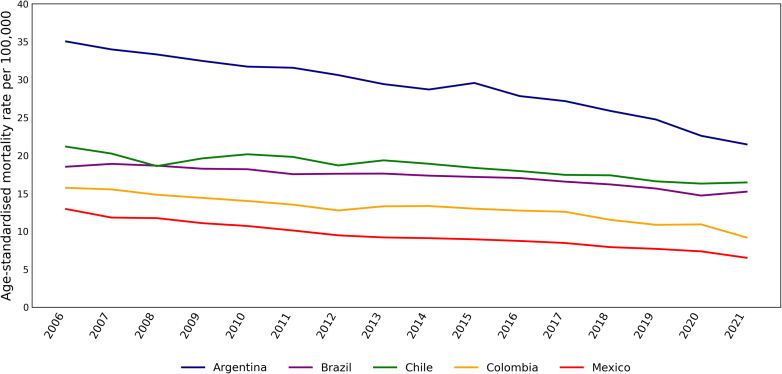
**Age-standardised mortality rate per 100,000 population related to tracheal, bronchus, and lung cancer in men between 2006 and 2021.** Source: This is an adaptation of an original work “WHO Mortality Database. Geneva: World Health Organization (WHO); [2022]. Licence: CC BY-NC-SA 3.0 IGO”. This adaptation was not created by WHO. WHO is not responsible for the content or accuracy of this adaptation. The original edition shall be the binding and authentic edition.^20^

Fig. 1 presents the age-standardised mortality rate per 100,000 population for lung cancer in men between 2006 and 2021. There was a reduction in the mortality rate consistent across the five countries, with a continuous decline over the years. Argentina had the most considerable age-standardised mortality rate reduction during the study period, with 13·6 deaths per 100,000 people. Brazil and Chile had the lowest reduction, with 7·2 and 6·6 deaths per 100,000, respectively. Despite having the lowest mortality rate at the beginning of the study, both Colombia and Mexico achieved a reduction of around 6·5 deaths per 100,000 by 2021.

The regression model results presented by country and age group, are shown in Supplementary Table S1. Overall, a significant positive effect of innovation on reducing lung cancer mortality was identified, primarily among adults over 40 years old. Notably, the impact of innovation on mortality was statistically significant in all five countries for the population aged between 45 and 59 years. Argentina was the country where a significant effect was found for most age groups (all above 35 years of age). The most significant effect size was found for Colombia, in the population between 75 and 79 years, where for each additional indication available in the country, mortality was reduced by 2·31 deaths per 100,000 people.

### Avoidable deaths

WHO reported 446,210 deaths due to lung cancer mortality in the five countries analysed between 2006 and 2021. Using the estimated impact of innovation on the mortality rate and the difference in approved therapies between the FDA and each country, we estimated 8694 potentially avoidable deaths if all innovations had been available locally. The results by country are presented in Table 1. Results by age group in each country are presented in Supplementary Table S2. Although the absolute number of potentially avoidable deaths is similar among most countries, this is relative to their population size, the country-specific lung cancer mortality rates, and the estimated impact of innovation on ASMR. The highest clinical impact, based on potentially avoided deaths, was found in Argentina, where lung cancer ASMR was the highest; still, a significant effect of innovation was observed in the majority of the population (Supplementary Table S2). In contrast, although Colombia does not have the highest ASMR nor the largest population, innovation has had a significant effect in reducing mortality, especially among the population over 75 years (Supplementary Table S2); thus, almost 2000 potentially avoidable deaths were estimated.

**Table 1:** Cumulative avoidable deaths, years of life lost, and productivity loss from delayed approval of innovative lung cancer treatments (2006–2021).

Country	Avoidable deaths	Years of life lost	Productivity loss
Argentina	2185 (871–3498)	30,446 (12,835–48,058)	165,316,883 (76,696,668–253,937,071)
Brazil	2014 (767–3261)	29,140 (10,130–48,150)	164,820,022 (57,739,624–271,900,444)
Chile	407 (215–600)	8032 (4216–11,847)	30,897,015 (15,909,222–45,884,813)
Colombia	1977 (580–3374)	26,425 (8845–44,005)	42,435,773 (20,955,023–63,916,526)
Mexico	2111 (1036–2870)	20,433 (10,303–30,564)	35,710,184 (17,063,038–54,357,380)
**Total**	**8694 (3469–13,603)**	**114,477 (46,329–182,625)**	**439,179,876 (188,363,576–689,996,234)**

The results in parentheses correspond to the results of the sensitivity analysis using the lower and upper limits of the confidence interval of the coefficient estimated in the regression model.

Results for each country by year are presented in Supplementary Table S3. Between 10% (218 in Mexico) and 31% (627 in Brazil) of the total avoidable deaths were estimated during the first decade of the analysis (2006–2015). This number increased to 41% (686 in Mexico) and to 51% (209 in Chile) between 2016 and 2019, consistent with the continuous development of innovative therapies that were not yet available in Latin American countries. Remarkably, between 28% (536 in Brazil) to 49% (1024 in Mexico) of all avoidable deaths were estimated between 2020 and 2021. Although this last period only included two years, the high number of potentially avoidable deaths could have been exacerbated by factors such as COVID-19, which could increase the delay in local approval of innovative treatments.

### Years of life lost

**Fig. 2:**
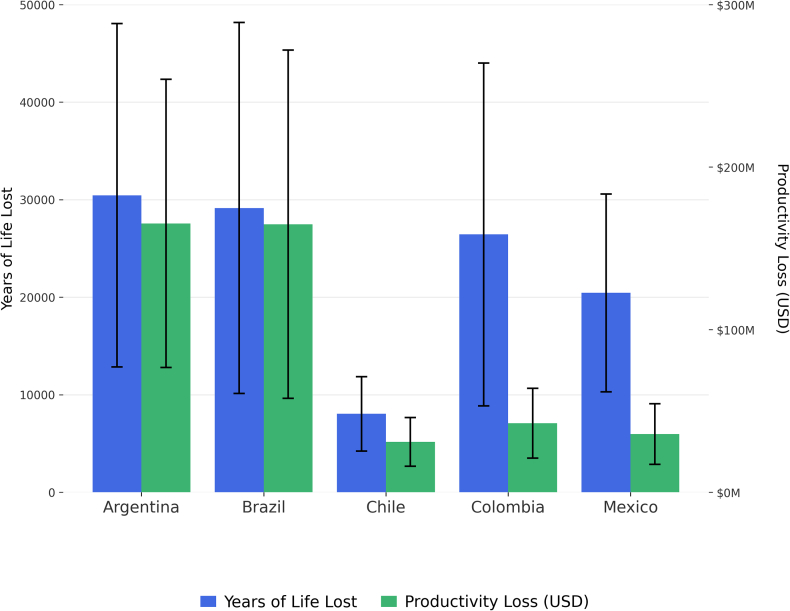
**Years of life lost and productivity loss from delayed approval of innovative lung cancer treatments.** Error bars represent the results of the sensitivity analysis using the lower and upper limits of the confidence interval of the coefficient estimated in the regression model.

The years of life lost were calculated based on the potentially avoidable deaths if the innovation had been available in each country without regulatory delays. A total of 114,477 years of life lost were estimated for the five countries included. Considering that the years of life lost were estimated from the total avoidable deaths, these also depend on population size, lung cancer mortality, and the effect of innovation in each country. As shown in Supplementary Table S2, in Argentina, innovation has shown a significant effect in reducing age-specific mortality in most age groups, allowing the years of life lost to be almost as high as those in Brazil despite having a significantly smaller population. Fig. 2 and Table 1 present the years of life lost per country. Although the prevalence and mortality were higher in patients over 60, a significant number of deaths were identified in the 40- to 64-year-old age group, who have a reduced life expectancy and generate a high number of years of life lost (Supplementary Table S2).

### Productivity loss

The productivity losses considered only the years of productive life based on each country’s labour force retirement age. The most significant productivity losses were found in Brazil and Argentina, mainly due to Brazil’s large population and Argentina’s high GDP per capita. Similarly, Chile has a high GDP per capita; therefore, despite having fewer years of life lost, it has a similar loss of productivity to other countries such as Mexico and Colombia, which have a high clinical impact but a lower GDP per capita. Results for productivity loss are presented in Table 1 and Fig. 2.

### Sensitivity analyses

The minimum and maximum values of each outcome reported in Table 1 represent the results of the sensitivity analysis based on the confidence interval for the effect of innovation available in each country on lung cancer mortality between 2006 and 2021 ($\beta_{1}$ of the regression model). This analysis continues to demonstrate a high clinical and economic impact related to the delay in local approval of the innovation for treating lung cancer in the region. The results reach more than three thousand avoidable deaths in Argentina, Brazil, and Colombia; nearly fifty thousand years of life lost in Argentina and Brazil; and productivity losses ranging from 45 to 270 million 2022-USD.

The results of the sensitivity analysis imputing the missing age-specific incidence of 2020 and 2021 with the latest reported value are presented in Supplementary Table S4. The results showed a slight variation in total potentially avoidable deaths, years of life lost, and productivity loss, with values between 1·5% (USD 432,458,683 in productivity loss) and 4·5% (8305 avoidable deaths) lower than the main analysis but within the minimum and maximum range presented in Table 1.

## Discussion

Our study assessed the impact of the availability of innovative treatments for advanced lung cancer in five Latin American countries. It showed an important clinical and economic benefit related to non-delayed approvals, significant gains in deaths, years of life lost, and productivity loss associated with early mortality that can be avoided through the timely approval of new therapies. The benefit of the innovation in reducing disease’s clinical impact is consistent with previous research. Our analyses identified a positive effect of innovation on the survival of adults between 40 and 60 years old for all included countries, highlighting the benefit of the availability of new therapies in reducing the disease burden related to early mortality from lung cancer. These findings are consistent with Lichtenberg’s study on the impact of new cancer drugs on clinical outcomes in 36 countries, where he found that in the absence of new launches between 2006 and 2010, there would have been 4·51 million additional years of life lost in patients under 75 years and 2·52 million in those under 65.^24^ In addition to the clinical impact, a negative economic effect related to high-burden diseases, such as cancer, has also been reported. The WifOR study on the socioeconomic of high-burden diseases in Latin America found that by 2022, neoplasms were responsible for an estimated loss of productivity between one and 6·6 billion in 2015-USD in the five countries included in our study.^26^ The WifOR study results indicate a significant burden of cancer in the working-age population, supporting the importance of our findings in highlighting, from an economic perspective, the substantial benefits of reducing cancer-related mortality in the working-age adult population that will translate into economic growth.

Additionally, innovation significantly benefits older populations. Lichtenberg et al. analysed the role of innovation in reducing years of life lost in older adults in 27 countries, concluding that if no innovative treatments were launched after 1981, the years of life lost in the population over 85 years would have been more than 2·16 times higher by 2013.^25^ The authors also found that these innovations saved 82·6 million life-years in the population under 70 years.^25^ While the analysis by Lichtenberg et al. was not unique to cancer, it highlights the impact of innovation on the elderly population. Our model found that available treatments positively affected the elderly populations, specifically in Argentina and Mexico; this positive effect of innovation was maintained in all adults from 40 to 85 years. Our results emphasise the benefits for the elderly population of receiving timely treatments, thereby reducing mortality and improving the population’s longevity, which has been described as an essential component of economic growth.^24^

Our study is based on the hypothesis that innovation in lung cancer treatment positively reduces mortality, but this should be interpreted with caution. For example, in countries such as Brazil, access to innovation has been limited, but cancer mortality has decreased, probably due to the reduction in cigarette consumption.^27^^,^^28^ Nevertheless, the relationship between the change in mortality and the availability of innovative therapies is supported by previous research.^3^^,^^10^ Howlader et al. found that the reduction in mortality in patients with NSCLC in the USA went from 3·2% in 2006 to 6·3% between 2013 and 2016.^10^ This accelerated reduction began in 2013 when recommendations for routine testing for *EGFR* and ALK alterations and the use of FDA-approved targeted therapies emerged, reducing mortality due to improved survival.^10^ However, as our study shows, there are significant delays in the local approval of new treatments for lung cancer, with treatments approved up to 5 years after FDA approval. Although variations among the five countries analysed, these delays meant more than 8000 deaths that could have been avoided with more timely approval processes, representing more than 110,000 years of life lost, and more than 400 million dollars in productivity losses.

An important finding of this study was that Argentina had the highest lung cancer mortality rate. This might be related to their high incidence of lung cancer, along with past and present high smoking rates,^29^ where, despite the implementation of anti-tobacco policies, 75% of teenagers begin smoking before the age of 14 years.^30^ Likewise, despite having a high molecular testing rate, in the public sector, access remains limited (41%).^30^ However, lung cancer mortality in Argentina has been decreasing over the past decades.^30^ This trend aligns with the findings in our study that showed that by 2021, Argentina had 70% of the FDA-approved therapies. Additionally, our model showed that innovation had a significant effect in reducing mortality in most age groups in this country. This supports the positive impact of innovation in reducing mortality, highlighting opportunities to improve lung cancer survival with timely approval of innovative treatments.

Nevertheless, is worth noting that marketing approval does not imply widespread use of the new molecule or a guarantee of access for the entire target population, as it does not automatically translate into incorporating these therapies into the public health system or reimbursement by private health companies. It often takes years for these drugs to become broadly available following regulatory approval. Lichtenberg found that there could be a lag of several years between the use of a new cancer drug and its impact on mortality.^24^ This reality may be exacerbated in Latin American countries, where it has been reported that the time between approval and broad reimbursement in countries such as Mexico and Chile can exceed 1000 days.^15^ These delays affect access to innovation because if a new drug does not reach a significant market share, it will not have the expected impact on survival.

These reimbursement delays can be attributed to the varying lengths of marketing authorisation and reimbursement decisions across different countries due to health technology assessment procedures and negotiations between manufacturers and national authorities.^31^ Our study did not consider reimbursement delays and focused on initial local regulatory approval; however, it targets an essential piece of the problem. By recognising and anticipating the potential delays in access due to the variability of reimbursement systems, improving regulatory approval times can reduce the overall impact on patients by ensuring adequate access to innovative treatments.

Recognising the relevance of developing strategies to enhance regulatory approval processes is critical. Some countries, such as France, have developed an Early Access Program to allow patients quicker access to innovative therapies before market approval. Other strategies include the FDA fast track, EMA prime, FDA priority review, FDA accelerated approval, FDA breakthrough therapy, and EMA conditional approval.^31^ One strategy implemented in Latin American countries to improve regulatory processes has been the regulatory reliance procedures, which can vary from considering and giving significant weight to evaluations performed by other agencies to granting direct approval or abbreviating the review process of a drug approved by a regulator from another jurisdiction.^32^^,^^33^

The formal reliance process adopted by each country may vary; for example, Argentina, Brazil, and Chile have a formal reliance pathway, but none of the five countries included in our analysis uses homologation strategies.^18^ In Argentina and Brazil, the implementation of reliance pathways and expedited reviews has shown some benefits in reducing approval times; however, these strategies have primarily focused on orphan drugs for rare diseases, and preliminary experiences have highlighted the need to continue adjusting and re-evaluating these strategies.^19^

However, the effect of these strategies has not yet been reflected in clinical outcomes or access to innovative medicines for lung cancer, highlighting that there are still opportunities to improve local regulatory processes, for example, by expanding the strategies implemented for rare diseases to other life-threatening diseases, or by implementing strategies that have proven to be effective, such as the programs for promising priority drugs used by the FDA and EMA.^18^ Additional strategies include implementing conditional approvals with requirement to generate additional evidence, or the consideration of more study designs, including real-world evidence, or clinical trials with adaptive designs,^18^ which allows the study to be modified based on preliminary results and have shown benefits by reducing the duration of the studies, and therefore, providing more efficient results for decision-making.^34^

An additional factor when analysing the availability of innovative therapies over time is the impact of the COVID-19 pandemic. According to the FIFARMA report, the regional availability of innovative therapies related to the FDA and EMA-approval years between 2017 and 2019 was 93%; however, between 2018 and 2021, regional availability dropped to 72%.^15^ The impact of COVID-19 and the challenges faced by healthcare systems in the region exacerbate delays in accessing innovative treatments,^15^ highlighting the importance of developing strategies that facilitate approval and access to innovation.

Our study has some limitations that must be acknowledged. Our projections of the number of potentially avoidable deaths, years of life lost, and productivity losses are based on a regression model that estimates the relationship between the availability of innovative treatments in each country and changes in mortality due to lung cancer. Regression model estimators are susceptible to omitted variable bias. Various factors can be related to mortality and treatment availability in a country, some of which may be unobservable; however, we attempted to overcome this limitation by including the age-specific incidence rate in the model. We consider the incidence rate a suitable control variable since it is highly related to possible omitted variables, including public health policies, health expenditure, and smoking patterns,^35^ which are challenging to measure and include in the regression model.

Our study assumes that all FDA-approved therapies will eventually be approved in the Latin American countries included. This assumption is supported by the historical approval data for lung cancer treatments analysed in the study, where even with delays of up to six years, FDA-approved treatments are eventually approved at the local level. Likewise, although our study was conducted up to 2021 due to the availability of local age-specific mortality data, therapies approved by the FDA in subsequent years have been approved locally, indicating that local approval continues to increase despite regulatory delays.

As previously mentioned, our analysis assumes that innovative therapies have a positive impact on reducing lung cancer mortality; however, no individual adjustment was made to the effectiveness of each therapy included. To make such a country-specific adjustment, real-world evidence for each innovative therapy in each country would be required; however, such evidence is often lacking. Nevertheless, evidence has demonstrated the impact of the increase in innovative therapies on reducing lung cancer mortality,^3^^,^^10^ supporting the assumption of our analysis.

Another limitation is that our study does not assess the cost to the health system of investing in innovative treatments for lung cancer, which is important since Latin American countries have limited resources and capacity in primary care, specialised oncology care, and palliative care.^29^ As discussed here, timely approval of innovative treatments improves survival outcomes; however, this implies a financial burden on healthcare systems. Although this is beyond the scope of our study, future research should estimate the healthcare economic impact of investing in innovative treatments.

Finally, our analysis was limited to the male population. This was due to the strong association between lung cancer mortality reduction and innovative therapies in males,^10^ which was the primary assumption of our analysis. For women, evidence has shown a lower decrease in lung cancer incidence compared to men, possibly associated with other variables such as exposure to indoor wood burning or differences in smoking prevalence by sex.^10^^,^^36^ It was considered that these variables may be so strongly associated with incidence and mortality in the female population that they need to be included as independent variables in the model. Future research may address this issue if sufficient data can be identified to adjust for confounding factors specific to the female population. Similarly, an analysis by ethnicity was not performed due to the absence of specific information on lung cancer mortality by ethnicity. The results of our study should be analysed considering each country’s population density and economic capacity, represented by their GDP per capita. Nevertheless, given that the data on lung cancer mortality in all five countries shows a reduction associated with the local entry of innovative treatments, our analysis highlights the importance of timely local approval as an opportunity to improve clinical and economic outcomes for a high-burden disease such as lung cancer.

Our study quantifies the clinical and economic impact of the lack of innovative treatments for advanced lung cancer in five Latin American countries, highlighting the importance of improving regulatory processes to increase the availability of innovative therapies. Accelerating the introduction of innovative therapies for lung cancer in Latin America represents a significant opportunity to enhance the population’s survival rates, instil a sense of hope and optimism, and avoid substantial productivity losses.

## Contributors

AC, LGB, NS, LPP, JZ, DS, AR and JK contributed to the study conception and design, interpretation of data and drafting of the manuscript. AC, LGB, NS, LPP, JZ, DS, AR and JK contributed to the acquisition and analysis of data. Statistical analysis was performed by AR, DSS, LPP. AC, LGB, NS, LPP and DS have directly accessed and verified the data contained in the manuscript. All authors contributed data or analysis tools and critical revision of the manuscript. All authors contributed in administrative, technical, or logistic support. AC and LPP were the study’s supervisors. All authors agreed with the final version of the manuscript to be published.

## Data sharing statement

All data relevant to the study and all data sources are included in the article or uploaded as Supplementary information.

## Declaration of interests

AC have declared grants/contracts from Merck Sharp & Dohme, Boehringer Ingelheim, Roche, Bristol-Myers, Squibb, Foundation Medicine, Roche Diagnostics, Termo Fisher, Broad Institute, Amgen, Flatiron Health, Teva Pharma, Rochem Biocare, Bayer, INQBox and The Foundation for Clinical and Applied Cancer Research – FICMAC. AC have also declared financial support for conferences and academic meetings, expert testimony, consulting fees and Advisory Boards from EISAI, Merck Serono, Jannsen Pharmaceutical, Merck Sharp & Dohme, Boehringer Ingelheim, Roche, Bristol-Myers Squibb, Pfizer, Novartis, Celldex Therapeutics, Foundation Medicine, Eli Lilly, Guardant Health, Illumina, and FICMAC. LGB has declared receiving honoraria for lectures, presentations, speakers’ bureaus, study writing or educational events from Janssen Cilag and Bristol Myers Squibb and support for attending meetings from MSD. JZ has declared receiving financial support for consulting fees and honoraria for lectures presentations, speakers’ bureaus, study writing or educational events from BMS, AstraZeneca, support for attending meetings from AstraZeneca, Tecnofarma and Pfizer, and materials, drugs, medical writing or other services from AstraZeneca, Roche and Pfizer. SM has declared receiving honoraria for lectures, medical writing and educational events from MSD, AstraZeneca, Medtronic and Johnson & Johnson. LV has declared receiving honoraria for lectures, presentations, speakers’ bureaus, study writing or educational events and support for attending meetings from MSD, Sanofi, AstraZeneca, Glaxo Smith-Kline and Brystol Myers Squibb. CC have declared to receive honoraria for lectures from AstraZeneca. CM has declared to receive honoraria for lectures presentations, speakers’ bureaus, study writing or educational events from Jannsen Cilag, AstraZeneca, MSD, BMS Takeda and Pfizer. LC has declared receiving financial support for consulting fees and honoraria for lectures presentations from Johnson and Johnson, MSD, AstraZeneca, Merck, Pfizer, GSK, BMS, and Novartis, as well as support for attending meetings from Johnson and Johnson, MSD, AstraZeneca, Pfizer and Novartis. LER declared grants/contracts from BMS, Lilly Oncology, Natera, Guardant Health, Genentech, Velos, BiAlta, Onc4, Pfizer, Bayer and Merck, and receiving consulting fees from AstraZeneca and BMS. VCL has declared financial support for conferences and academic meetings, and for consulting fees and Advisory Boards from Cristália, Roche, Janssen, Merck Serono, BMS, MSD and Astrazeneca in Brazil. SS has declared receiving honoraria for lectures, presentations, speakers’ bureaus, study writing or educational events from AstraZeneca, Pfizer, BMS and Roche, as well as support for attending meetings from Roche, Merck, AstraZeneca, Pfizer and BMS. OA have declared receiving grants/contracts from AstraZeneca, Boehringer Ingelheim, and Roche, and consulting fees from Pfizer, Eli Lilly, Merck, Bristol Myers Squibb, AstraZeneca, Boehringer Ingelheim, and Roche. DS-S, P-PL and KJ are employees of Roche, Colombia. KJ declared participation in the Roche employee stock purchase program. AR was an employee of Roche Colombia at the time of the study. NS, LB and LR have declared no conflict of interest. None of the authors received any compensation for the authorship of this manuscript.

## Appendix ASupplementary data

Supplementary Tables S1–S4

Translated Summary_disclaimer
