# Supplementary material for: Clinical and economic impact of the availability of innovative therapies for advanced lung cancer in men in Latin America: a population-based secondary data study
Source: Lancet Reg Health Am. 2025 Jul 2;49:101172. doi: 10.1016/j.lana.2025.101172 (PMC12270047; doi:10.1016/j.lana.2025.101172)
Supplement: Supplementary Tables S1–S4 [file mmc1.pdf]

# Clinical and economic impact of the availability of innovative therapies for advanced lung cancer in men in Latin America

*Supplementary material*

Supplementary Table 1. Impact of innovation on lung cancer mortality of the number of available innovations per country ........ 2
Supplementary Table 2. Avoidable deaths (AD) and years of life lost (YLL) by age group and country ........ 3
Supplementary Table 3. Avoidable deaths, years of life lost and productivity lost by year and country. ........ 4
Supplementary Table 4. Sensitivity analysis - incidence for 2020 and 2021 with imputation of the last reported value (2019). ........ 7

*Supplementary Table 1. Impact of innovation on lung cancer mortality of the number of available innovations per country*

| Age group | Argentina | Brazil | Chile | Colombia | Mexico |
|---|---|---|---|---|---|
| [15-19] | **-0.006†**<br>**(-0.009, -0.002)** | -0.003<br>(-0.006, 0.000) | -0.003<br>(-0.010, 0.005) | -0.008<br>(-0.022, 0.006) | **-0.003†**<br>**(-0.005, -0.0002)** |
| [20-24] | -0.003<br>(-0.011, 0.005) | 0.002<br>(-0.001, 0.005) | -0.006<br>(-0.017, 0.006) | -0.004<br>(-0.016, 0.007) | 0.001<br>(-0.004, 0.005) |
| [25-29] | -0.003<br>(-0.020, 0.015) | -0.001<br>(-0.004, 0.001) | 0.003<br>(-0.004, 0.011) | **-0.019†**<br>**(-0.028, -0.009)** | 0.000<br>(-0.004, 0.004) |
| [30-34] | 0.066<br>(-0.043, 0.175) | 0.037<br>(-0.034, 0.108) | -0.014<br>(-0.032, 0.003) | -0.010<br>(-0.025, 0.005) | -0.004<br>(-0.009, 0.001) |
| [35-39] | **-0.031†**<br>**(-0.051, -0.010)** | -0.005<br>(-0.012, 0.001) | 0.002<br>(-0.056, 0.059) | 0.007<br>(-0.050, 0.063) | 0.000<br>(-0.027, 0.027) |
| [40-44] | **-0.138†**<br>**(-0.186, -0.090)** | **-0.058†**<br>**(-0.089, -0.026)** | **-0.042†**<br>**(-0.077, -0.007)** | -0.025<br>(-0.056, 0.006) | **-0.031†**<br>**(-0.060, -0.003)** |
| [45-49] | **-0.459†**<br>**(-0.709, -0.209)** | **-0.152†**<br>**(-0.216, -0.089)** | **-0.140†**<br>**(-0.186, -0.093)** | **-0.174†**<br>**(-0.240, -0.107)** | **-0.078†**<br>**(-0.110, -0.047)** |
| [50-54] | **-1.097†**<br>**(-1.697, -0.496)** | **-0.173†**<br>**(-0.329, -0.017)** | **-0.275†**<br>**(-0.430, -0.119)** | **-0.347†**<br>**(-0.537, -0.158)** | **-0.156†**<br>**(-0.237, -0.075)** |
| [55-59] | **-1.680†**<br>**(-2.581, -0.779)** | **-0.331†**<br>**(-0.592, -0.069)** | **-0.637†**<br>**(-0.923, -0.351)** | **-0.507†**<br>**(-0.812, -0.202)** | **-0.315†**<br>**(-0.457, -0.173)** |
| [60-64] | **-1.541†**<br>**(-2.512, -0.570)** | -0.297<br>(-0.707, 0.112) | **-1.108†**<br>**(-1.605, -0.611)** | **-0.568†**<br>**(-1.059, -0.076)** | **-0.578†**<br>**(-0.833, -0.324)** |
| [65-69] | **-2.024†**<br>**(-3.328, -0.720)** | -0.245<br>(-0.628, 0.138) | **-1.392†**<br>**(-2.071, -0.713)** | -0.918<br>(-1.871, 0.035) | **-0.997†**<br>**(-1.538, -0.455)** |
| [70-74] | **-1.894†**<br>**(-3.324, -0.464)** | -0.461<br>(-1.141, 0.219) | -0.077<br>(-0.621, 0.466) | **-1.377†**<br>**(-2.563, -0.192)** | **-1.813†**<br>**(-2.720, -0.906)** |
| [75-79] | **-1.644†**<br>**(-2.790, -0.499)** | **-1.187†**<br>**(-1.677, -0.697)** | -0.139<br>(-0.894, 0.616) | **-2.312†**<br>**(-4.102, -0.522)** | **-1.983†**<br>**(-2.694, -1.273)** |
| [80-84] | **-1.684†**<br>**(-2.426, -0.942)** | -0.347<br>(-1.178, 0.483) | 0.353<br>(-0.667, 1.373) | **-2.222†**<br>**(-3.779, -0.666)** | **-1.794†**<br>**(-2.591, -0.998)** |
| [85+] | **-1.811†**<br>**(-2.824, -0.798)** | -0.611<br>(-3.809, 2.587) | 0.211<br>(-1.266, 1.689) | -1.129<br>(-3.187, 0.929) | **-1.782†**<br>**(-2.719, -0.845)** |

† Statistical significance ($p < 0.05$)

*Supplementary Table 2. Avoidable deaths (AD) and years of life lost (YLL) by age group and country*

| Age group | Argentina | | Brazil | | Chile | | Colombia | | Mexico | |
|---|---|---|---|---|---|---|---|---|---|---|
| | AD | YLL | AD | YLL | AD | YLL | AD | YLL | AD | YLL |
| [15-19] | 2 | 139 | - | - | - | - | - | - | 3 | 162 |
| [20-24] | - | - | - | - | - | - | - | - | - | - |
| [25-29] | - | - | - | - | - | - | 22 | 983 | - | - |
| [30-34] | - | - | - | - | - | - | - | - | - | - |
| [35-39] | 12 | 426 | - | - | - | - | - | - | - | - |
| [40-44] | 50 | 1,546 | 147 | 4,265 | 7 | 252 | - | - | 27 | 731 |
| [45-49] | 142 | 3,685 | 348 | 8,331 | 23 | 680 | 137 | 3,486 | 62 | 1,345 |
| [50-54] | 295 | 6,220 | 356 | 6,768 | 41 | 1,041 | 259 | 5,305 | 107 | 1,775 |
| [55-59] | 411 | 6,594 | 587 | 8,209 | 86 | 1,721 | 330 | 5,096 | 177 | 2,063 |
| [60-64] | 333 | 5,565 | - | - | 125 | 2,482 | 291 | 5,878 | 260 | 4,804 |
| [65-69] | 364 | 4,264 | - | - | 125 | 1,855 | - | - | 344 | 4,623 |
| [70-74] | 261 | 1,750 | - | - | - | - | 355 | 3,639 | 446 | 3,773 |
| [75-79] | 151 | 258 | 576 | 1,567 | - | - | 379 | 1,982 | 335 | 1,158 |
| [80-84] | 91 | - | - | - | - | - | 204 | 57 | 192 | - |
| [85+] | 71 | - | - | - | - | - | - | - | 158 | - |
| Total | 2,185 | 30,446 | 2,014 | 29,140 | 407 | 8,032 | 1,977 | 26,425 | 2,111 | 20,433 |

*Supplementary Table 3. Avoidable deaths, years of life lost and productivity lost by year and country.*

| Avoidable deaths | | | | | |
|---|---|---|---|---|---|
| **Year** | **Argentina** | **Brazil** | **Chile** | **Colombia** | **Mexico** |
| 2006 | - | - | - | - | - |
| 2007 | - | 44 (17-70) | 11 (6-16) | 24 (7-40) | 65 (32-88) |
| 2008 | - | 45 (17-73) | 11 (6-17) | - | 67 (33-91) |
| 2009 | - | - | 12 (6-18) | - | - |
| 2010 | - | - | 12 (6-18) | - | - |
| 2011 | - | - | - | - | - |
| 2012 | - | 50 (19-82) | - (7-19) | 29 (9-49) | - |
| 2013 | - | 52 (20-84) | - | 30 (9-51) | - |
| 2014 | 85 (34-135) | 161 (61-260) | - | 94 (28-160) | - |
| 2015 | 172 (69-275) | 275 (104-446) | - | 130 (39-221) | 86 (42-117) |
| 2016 | 175 (70-280) | 170 (64-275) | 30 (16-45) | 135 (40-230) | - |
| 2017 | 178 (71-284) | 232 (88-376) | 63 (33-92) | 176 (52-299) | 279 (136-378) |
| 2018 | 361 (144-578) | 119 (45-192) | 16 (9-24) | 256 (75-437) | 290 (142-392) |
| 2019 | 367 (146-588) | 304 (116-491) | 100 (53-147) | 381 (111-652) | 299 (147-406) |
| 2020 | 280 (112-448) | 248 (95-401) | 68 (36-101) | 356 (103-608) | 305 (150-415) |
| 2021 | 567 (226-908) | 315 (121-510) | 70 (37-103) | 366 (105-626) | 719 (354-983) |
| Total | 2185 (871-3498) | 2014 (767-3261) | 407 (215-600) | 1977 (580-3374) | 2111 (1036-2870) |
| **Years of life lost** | | | | | |
| **Year** | **Argentina** | **Brazil** | **Chile** | **Colombia** | **Mexico** |
| 2006 | - | - | - | - | - |
| 2007 | - | 574 (204-944) | 197 (103-291) | 286 (104-467) | 652 (325-978) |
| 2008 | - | 605 (214-997) | 207 (108-305) | - | 672 (336-1007) |
| 2009 | - | - | 215 (112-318) | - | - |
| 2010 | - | - | 230 (120-339) | - | - |
| 2011 | - | - | - | - | - |
| 2012 | - | 715 (250-1180) | 250 (131-368) | 390 (138-642) | - |
| 2013 | - | 749 (260-1238) | - | 408 (144-671) | - |
| 2014 | 1180 (500-1860) | 2349 (809-3889) | - | 1275 (448-2101) | - |
| 2015 | 2413 (1019-3807) | 4040 (1401-6680) | - | 1795 (624-2966) | 874 (440-1308) |

| | | | | | |
|---|---|---|---|---|---|
| 2016 | 2423 (1021-3824) | 2472 (855-4088) | 601 (315-886) | 1868 (646-3090) | - |
| 2017 | 2503 (1057-3949) | 3453 (1189-5717) | 1251 (657-1845) | 2431 (836-4026) | 2792 (1407-4177) |
| 2018 | 5100 (2155-8045) | 1789 (615-2962) | 320 (168-472) | 3530 (1206-5854) | 2886 (1455-4317) |
| 2019 | 5253 (2218-8289) | 4648 (1606-7689) | 2029 (1066-2992) | 5327 (1787-8867) | 3011 (1519-4502) |
| 2020 | 3849 (1619-6079) | 3523 (1232-5814) | 1356 (712-2000) | 4642 (1508-7777) | 2838 (1433-4243) |
| 2021 | 7725 (3246-12204) | 4222 (1493-6951) | 1377 (724-2031) | 4474 (1405-7544) | 6709 (3388-10031) |
| Total | 30446 (12835-48058) | 29140 (10130-48150) | 8032 (4216-11847) | 26425 (8845-44005) | 20433 (10303-30564) |
| **Productivity loss** | | | | | |
| **Year** | **Argentina** | **Brazil** | **Chile** | **Colombia** | **Mexico** |
| 2006 | - | - | - | - | - |
| 2007 | - | 3,480,251 (1,262,489-5,698,014) | 780,237 (399,197-1,161,276) | 468,868 (235,918-701,817) | 1,138,771 (531,038-1,746,505) |
| 2008 | - | 3,726,892 (1,347,579-6,106,206) | 826,592 (423,271-1,229,913) | - | 1,175,106 (549,138-1,801,076) |
| 2009 | - | - | 833,138 (427,018-1,239,258) | - | - |
| 2010 | - | - | 898,100 (460,779-1,335,421) | - | - |
| 2011 | - | - | - | - | - |
| 2012 | - | 4,494,992 (1,597,603-7,392,383) | 1,045,970 (537,607-1,554,333) | 634,447 (317,087-951,807) | - |
| 2013 | - | 4,686,756 (1,656,391-7,717,121) | - | 677,495 (337,876-1,017,113) | - |
| 2014 | 6,840,579 (3,164,654-10,516,503) | 14,293,261 (5,024,082-23,562,443) | - | 2,152,133 (1,070,742-3,233,524) | - |
| 2015 | 14,096,515 (6,525,895-21,667,132) | 23,228,054 (8,128,741-38,327,371) | - | 2,986,463 (1,482,142-4,490,784) | 1,518,045 (720,387-2,315,704) |
| 2016 | 13,848,778 (6,416,071-21,281,482) | 13,627,561 (4,754,925-22,500,198) | 2,390,493 (1,231,546-3,549,440) | 3,073,948 (1,521,895-4,626,001) | - |
| 2017 | 14,292,725 (6,626,821-21,958,628) | 18,613,427 (6,485,328-30,741,529) | 4,871,764 (2,510,433-7,233,095) | 3,912,598 (1,933,032-5,892,164) | 4,898,999 (2,335,467-7,462,539) |
| 2018 | 27,972,660 (12,978,676-42,966,639) | 9,576,599 (3,337,150-15,816,051) | 1,270,868 (654,876-1,886,860) | 5,626,077 (2,775,059-8,477,096) | 5,089,875 (2,431,322-7,748,435) |
| 2019 | 27,583,735 (12,805,161-42,362,303) | 24,498,421 (8,546,314-40,450,532) | 7,703,195 (3,969,159-11,437,233) | 8,295,005 (4,086,947-12,503,064) | 5,165,389 (2,471,943-7,858,842) |
| 2020 | 18,773,721 (8,717,891-28,829,549) | 19,166,428 (6,696,536-31,636,323) | 4,840,895 (2,494,383-7,187,408) | 6,924,418 (3,409,821-10,439,015) | 4,781,581 (2,291,834-7,271,334) |

| | | | | | |
|---|---|---|---|---|---|
| 2021 | 41,908,170 (19,461,498-64,354,834) | 25,427,378 (8,902,487-41,952,273) | 5,435,764 (2,800,953-8,070,576) | 7,684,322 (3,784,504-11,584,141) | 11,942,419 (5,731,909-18,152,944) |
| Total | 165,316,883 (76,696,668-253,937,071) | 164,820,022 (57,739,624-271,900,444) | 30,897,015 (15,909,222-45,884,813) | 42,435,773 (20,955,023-63,916,526) | 35,710,184 (17,063,038-54,357,380) |

*Supplementary Table 4. Sensitivity analysis - incidence for 2020 and 2021 with imputation of the last reported value (2019).*

| Country | Avoidable deaths | Years of life lost | Productivity loss |
|---|---|---|---|
| **Argentina** | 2,097 | 29,963 | 166,980,994 |
| **Brazil** | 1,902 | 28,180 | 159,904,606 |
| **Chile** | 393 | 7,759 | 29,503,315 |
| **Colombia** | 1,835 | 24,681 | 40,652,830 |
| **Mexico** | 2,078 | 20,105 | 35,416,938 |
| **TOTAL** | **8,305** | **110,688** | **432,458,683** |
