# Supplementary material for: Clinical and economic impact of the availability of innovative therapies for advanced lung cancer in men in Latin America: a population-based secondary data study
Source: Lancet Reg Health Am. 2025 Jul 2;49:101172. doi: 10.1016/j.lana.2025.101172 (PMC12270047; doi:10.1016/j.lana.2025.101172)
Supplement: Translated Summary_disclaimer [file mmc2.docx]

*Editor note: This translation in Spanish was submitted by the authors and we reproduce it as supplied. It has not been peer reviewed. Our editorial processes have only been applied to the original abstract in English, which should serve as reference for this manuscript.*

**Translated Summary (Spanish):**

**Antecedentes**: Durante la última década, el desarrollo de terapias innovadoras contra el cáncer se ha acelerado y ha sido asociado con una disminución de la mortalidad por cáncer; sin embargo, los tiempos de aprobación regulatoria local son extensos. Este estudio estimó el impacto clínico y económico de las demoras en la aprobación de terapias innovadoras para el tratamiento del cáncer de pulmón avanzado en hombres en cinco países de Latinoamérica.

**Métodos:** Utilizando datos públicos, se estimó la relación entre las terapias innovadoras disponibles (TID) y la tasa de mortalidad específica por edad (TME) para Argentina, Brasil, Chile, Colombia y México a través de un modelo de regresión. A partir de la diferencia entre el número de terapias aprobadas por la FDA y el número aprobado por cada agencia local, se calcularon las muertes evitables (ME) si la innovación hubiera estado disponible. Los Años de Vida Perdidos (AVP) se estimaron utilizando la esperanza de vida, la edad mediana de muerte y las ME. La pérdida de productividad (PP) se calculó utilizando la edad de jubilación de cada país y el Producto Interno Bruto per cápita anual en dólares estadounidenses constantes de 2022.

**Resultados:** El total de ME, AVP y PP fue de 8,694, 114,477 y USD 439,179,876, respectivamente. Argentina tuvo el mayor impacto de las TID en la TME. Los resultados de Brasil mostraron un alto impacto clínico y económico, principalmente debido a su gran población, mientras que el alto PIB per cápita de Chile condujo a una alta PP. Colombia y México mostraron un alto impacto clínico, lo que sugiere un beneficio de la aprobación temprana. Las diferencias en la disponibilidad y los tiempos de aprobación han incrementado con el número de terapias aprobadas por la FDA; sin embargo, las brechas de tiempo para aprobación local han aumentado recientemente.

**Interpretación:** Nuestro estudio muestra el sustancial impacto clínico y económico de las demoras en la aprobación de terapias innovadoras, lo que resalta el potencial de mejorar los procesos regulatorios para aumentar la disponibilidad de tratamientos contra el cáncer de pulmón. Acelerar la introducción de terapias innovadoras para el cáncer de pulmón avanzado en Latinoamérica representa una oportunidad significativa para mejorar las tasas de supervivencia, infundiendo esperanza y optimismo, al tiempo que se evitan pérdidas de productividad sustanciales.

**Financiación:** Este estudio se llevó a cabo como parte de un acuerdo de investigación entre Roche y CTIC. No se recibió financiación.
